# An Elusive Diagnosis of Autosomal Dominant Alport Syndrome: Genomic Sequencing Is a Game Changer

**DOI:** 10.7759/cureus.94077

**Published:** 2025-10-07

**Authors:** Achilleas Betsikos, Eleni Paschou, Virginia Geladari, Evanthia Gazouni, Nikolaos Sabanis

**Affiliations:** 1 1st/2nd Department of Internal Medicine, General Hospital of Trikala, Trikala, GRC; 2 Department of General Practice and Family Medicine, 10th Local Medical Unit of Giannouli, Larissa, GRC; 3 1st Department of Internal Medicine, General Hospital of Trikala, Trikala, GRC; 4 Department of Nephrology, General Hospital of Trikala, Trikala, GRC

**Keywords:** alport syndrome, benign familial hematuria, c.1321_1369+3del mutation, thin basement membrane disease, type-iv collagen diseases

## Abstract

We report the case of a 55-year-old woman with chronic asymptomatic hematuria and a longstanding diagnosis of thin basement membrane disease, presenting with worsening hypertension and a significant degree of proteinuria progressing to chronic kidney disease. Genetic sequencing identified a heterozygous pathogenic variant in the COL4A4 gene (c.1321_1369+3del), a 52-base pair deletion that disrupts normal ribonucleic acid (RNA) splicing in the exon 20/intron 20 junction. In silico analysis predicted the complete loss of the canonical splice site, leading to improper splicing and the dysfunction of the α4 chain of type-IV collagen. Genetic testing in the family confirmed the presence of the same variant in two additional generations. To our knowledge, this is the first reported family in which the c.1321_1369+3del mutation is the sole cause of autosomal dominant Alport syndrome.

## Introduction

Alport syndrome and thin basement membrane nephropathy (previously known as benign familial hematuria) are inherited disorders of basement membrane collagen with a common molecular basis. More specifically, these conditions arise from mutations in genes COL4A3, COL4A4, and COL4A5, responsible for the production of α3, α4, and α5 chains of type-IV collagen, respectively, the major component of the mature glomerular basement membrane, that can be transmitted in an X-linked, autosomal recessive, or autosomal dominant pattern, leading to a heterogeneous array of clinical phenotypes [1].

On the basis of the above shared molecular cause of Alport syndrome and thin basement membrane nephropathy, these two entities are now classified as forms of collagen IV-related renal diseases [2]. Certainly, a unified classification of genetic disorders of collagen IV may substantially increase the number of affected individuals with Alport syndrome, which was estimated at approximately 1 in 5,000-10,000 people in the general population in the United States and is currently estimated to affect approximately 3% of children with chronic kidney disease and 0.2% of adults with end-stage renal disease, according to the ORPHANET organization. 

The classical presentation of Alport syndrome, which follows the X-linked pattern of inheritance, one of the oldest recognized hereditary nephropathies, has traditionally been defined as the clinical triad of glomerular renal disease leading to chronic kidney disease, ocular abnormalities, and bilateral sensorineural hearing loss. Similarly, individuals with autosomal recessive Alport syndrome follow an unfavorable clinical course, whereas individuals with an autosomal dominant pattern of inheritance manifest a highly variable clinical phenotype [2].

Thus, Alport syndrome is now widely viewed as a spectrum of diseases affecting type IV collagen and characterized by a broad clinical variability ranging from asymptomatic hematuria without extrarenal manifestations to full-blown classical Alport syndrome [3].

In this context, we aim to present a family with Alport syndrome due to a novel heterozygous pathological variant involving the type-IV collagen alpha-4 chain gene (COL4A4) following the autosomal dominant pattern of inheritance. We also analyze the potential implications of the C.1321_1369+3del mutation on a molecular basis, given that the same pathogenic variant has been associated only with thin basement membrane nephropathy in previous reports.

## Case presentation

A 55-year-old woman presented as an outpatient to the Nephrology Department of this hospital complaining about worsening hypertension. She was alert and calm. On auscultation, lung and heart sounds were normal. There was neither pedal edema nor periorbital edema. Clinical examination was completely unremarkable. The blood pressure was 155/85 mmHg, the heart rate 73 beats per minute, and the oxygen saturation 98% while the patient was breathing ambient air. The patient’s medical history was notable for primary hyperparathyroidism, dyslipidemia, arterial hypertension, and ANA-positive carpometacarpal arthritis. She noted experiencing recurrent mouth mucosal infections despite having a tonsillectomy. Medications included ramipril, nebivolol, hydroxychloroquine, omeprazole, allopurinol, rosuvastatin, cinacalcet, and vitamin D supplementation. There were no allergies reported.

Twelve years before the current presentation, this woman was evaluated for chronic asymptomatic microscopic hematuria. She underwent a renal biopsy. She received a diagnosis of thin basement membrane disease (formerly also known as benign familial hematuria) on the basis of incomplete type-IV collagen’s alpha-5 chain staining in immunohistochemistry (described as “signs of Alport’s glomerulopathy”). She was prescribed ramipril 2.5 mg daily. Furthermore, there was a positive family history of renal disease. Her brother progressed to end-stage kidney disease at the age of 45 due to focal and segmental glomerulosclerosis. The woman’s daughter also reported asymptomatic microscopic hematuria. One year before the current presentation, this woman’s granddaughter, aged six then, also reported microscopic hematuria. Taken all together, the family was offered genetic counseling, and consequently, genetic testing on the granddaughter was decided (Next Generation Sequencing). The results were negative for pathogenic variants in genes responsible for Alport’s syndrome or thin basement membrane disease.

As the first step in our current evaluation, a comprehensive assessment was initiated, which included a renal ultrasound to assess kidney structure, a series of laboratory tests, urinalysis, and a 24-hour urine collection to assess proteinuria. The results were notable for 711 mg of protein in the sample, while microscopic urinalysis identified dysmorphic red blood cells. More laboratory tests are available in Table 1.

**Table 1:** Laboratory tests

Variables	Patient	Reference Range
White Blood Cells (10^3^/μL)	5.11	4-10.8
Hematocrit/Hemoglobin (%, g/dL)	43.2/14.9	37.7-47.9/11.8 -17.8
Platelets(10^3^/μL)	271	150-350
Albumin (g/dL)	3.9	3.4-4.8
Urea (mg/dL)	29	10-50
Creatinine(mg/dL)	0.90	0.40-1.1
Sodium (mEq/L)	144	136-145
Potassium(mEq/L)	4.48	3.5-5.1
Serum Glutamic-Oxaloacetic Transaminase (IU/L)	28	5-40
Serum Glutamic Pyruvic Transaminase (IU/L)	31	10-37
Lactic Dehydrogenase (IU/L)	201	135-225
Creatine phosphokinase (IU/L)	113	24-190
Calcium(mg/dL)	10	8.1-10.4
C-Reactive Protein (mg/dL)	0.2	<0.7
Concentration of glycated hemoglobin A1 (HbA1C) (%)	5.6	4-6%
Alkaline Phosphatase (IU/L)	51	44-147
gamma-Glutamyl Transferase (γ-GT) (IU/L)	18	5-40
Vitamin D (ng/mL)	18.6	>30
intact- Parathyroid Hormone/ iPTH (pg/mL)	89	17-88
Magnesium(mg/dL)	2	1.6-2.3
Vitamin B12 (pg/mL)	821	200-900
Follic acid (ng/mL)	3.21	2.7-17
24-hour urine total protein	711 / 508	<150mg/24-hour
anti-double stranded DNA (anti-dsDNA)	Negative	
Antineutrophil Cytoplasmic Antibodies (ANCA)	Negative	
Complement Factor 3 (C3) (mg/dL)	145	88-201
Complement Factor 4 (C4) (mg/dL)	29	15-45
Antinuclear Antibodies (ANA)	Positive. 1/320	

**Figure 1:**
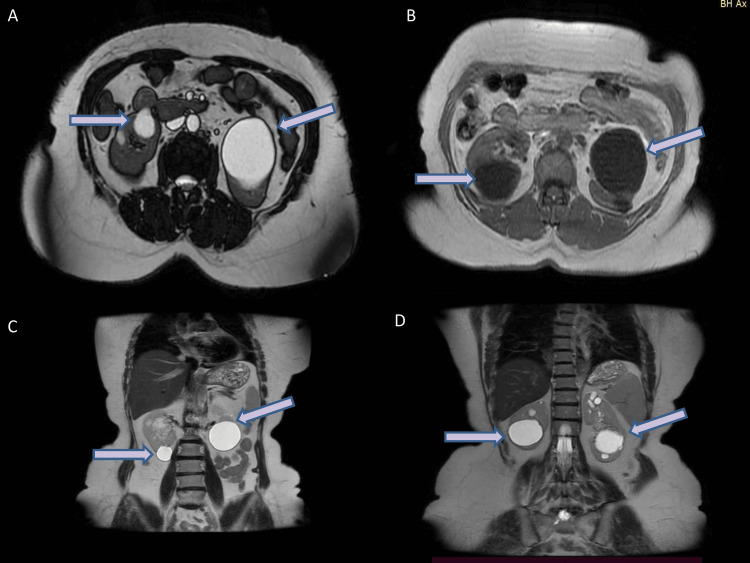
Magnetic resonance imaging of the abdomen Axial (A, B) and coronal (C, D) T2-weighted MRI images show normal-sized kidneys and several round, thin-walled, homogeneous, fluid-filled cysts (arrows), distributed throughout the renal parenchyma and located predominantly in the renal cortex.

Renal ultrasound demonstrated the presence of cysts in both kidneys. Ramipril was increased to 5 mg daily. We decided to proceed with an abdominal MRI, fundoscopy, and optical acuity testing, as well as an audiogram. Ophthalmology and otolaryngology were called; investigations yielded normal findings. MRI of the abdomen also revealed renal cysts in both kidneys (Bosniak II), in accordance with the aforementioned ultrasound (Figure 1).

We repeated the 24-hour urine collection only to find improvement in proteinuria levels after one month of follow-up (508 mg/24h).

**Figure 2:**
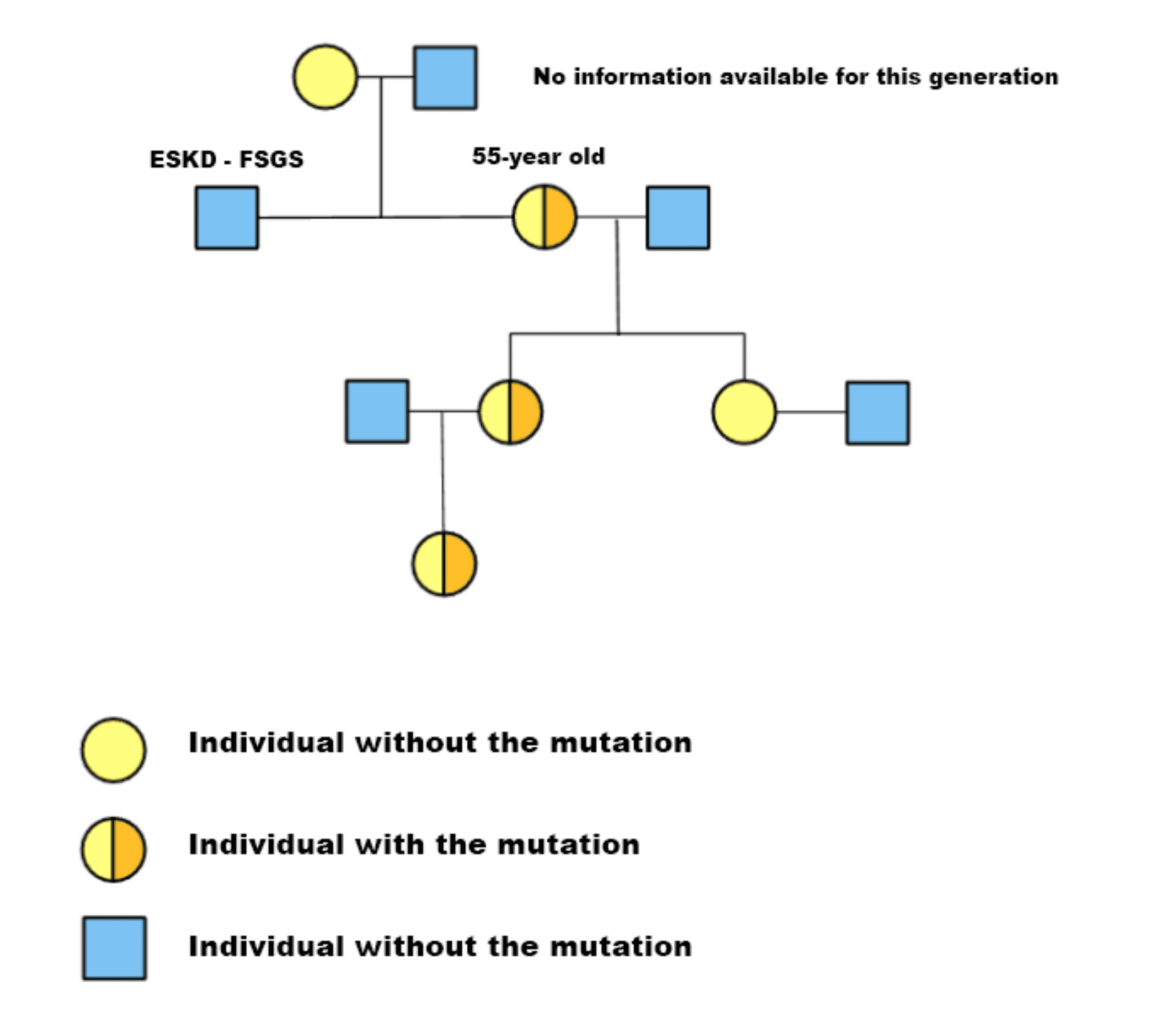
The pedigree chart of this family The pedigree of this family is depicted as a diagram revealing the presence of the C.1321_1369+3del heterozygous pathological variant in three generations of affected individuals. The image is created by the author.

Despite the fact that thin basement membrane disease can account for mild proteinuria, this patient’s sustained abnormally high levels of protein in the urine suggested renal pathology, which could no longer be considered benign. More than that, the presence of multiple simple cysts in both kidneys, located predominantly in the renal cortex, raises the suspicion of another underlying kidney disease, given that Alport syndrome with a cystic phenotype is an alternative cause for kidney cysts beyond Autosomal Dominant Polycystic Kidney Disease (ADPKD). Ramipril was increased to 7.5 mg daily in combination with a Sodium-Glucose Transport Protein 2 (SGLT2) inhibitor (dapagliflozin) at a dose of 10 mg daily. We proposed to proceed with genetic testing. The family agreed, and genomic sequencing in this woman was performed. A heterozygous pathological variant involving the type-IV collagen alpha-4 chain gene (COL4A4) was discovered; specifically, C.1321_1369+3del. Subsequent genetic testing of her daughter and retesting of her granddaughter identified the exact same heterozygous variant, while her brother refused. The pedigree of this family is depicted as a diagram in Figure 2.

## Discussion

From 1927, the year in which Cecil Alport first described Alport syndrome [4], until today, almost 100 years later, humanity’s perception of medicine has radically changed. What constituted a disease then was merely an ambiguous collection of observations, whereas in the present, we have shifted to well-described entities of genetics, laboratories, and imaging, as well as clinical findings. Nowadays, Alport syndrome is considered a spectrum of diseases affecting type IV collagen, with complex genetic interplay, rather than a monogenic disorder strictly following the principles of Mendelian inheritance, as was previously thought [5].

Alport syndrome was classically defined as an X-linked hereditary disorder resulting from alterations in type IV collagen that give rise to the clinical triad of kidney disease, sensorineural hearing loss, and ocular disease. It is now known that variable inheritance patterns are at play, including autosomal dominant and recessive forms, along with digenic and additive inheritance. Type IV collagen is a crucial structural protein of the glomerular basement membrane. Mutations in COL4A3, COL4A4, and COL4A5 encoding the α3, α4, and α5 chains of type IV collagen underlie the pathogenesis of Alport syndrome [2]. Special mention must be made of the Thin Basement Membrane Disease (formerly termed Benign Familial Hematuria), characterized by asymptomatic hematuria and mild albuminuria, albeit without progression to chronic kidney disease. Traditionally, a separate entity due to its previously thought benign course, it is now recognized as a manifestation of Alport syndrome [6].

**Figure 3:**
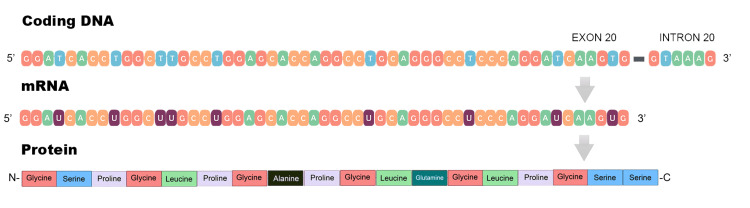
The gene COL4A4 (OMIM: 120131) is found on chromosome 2 and encodes the α4 chain of type IV collagen The gene COL4A4 is located on chromosome 2 and encodes the α4 chain of type IV collagen. The designed representation of this process was made through the NCBI Genome Data Viewer of the genetic sequence of interest (allele description: rs1553676221/NM_000092.4/COL4A4) by Achilleas Betsikos and Nikolaos Sabanis.

In this case, we present the molecular basis of the C.1321_1369+3del mutation and the potential implications it bears. For the following analysis, we used NCBI Genome Data Viewer to obtain the genetic sequence of interest (allele description: rs1553676221/NM_000092.4/COL4A4). The gene COL4A4 (OMIM: 120131) is found on chromosome 2 and encodes the α4 chain of type IV collagen (Figure 3).

**Figure 4:**
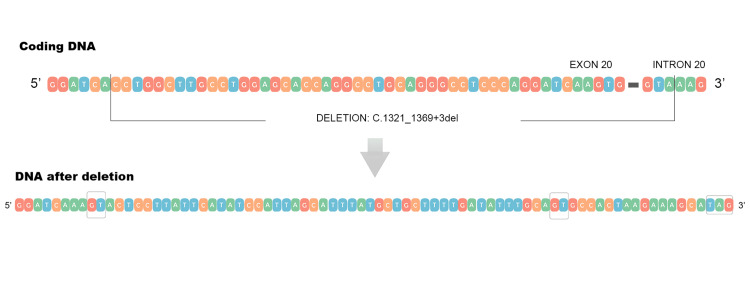
The molecular basis of the C.1321_1369+3del mutation The molecular basis of the C.1321_1369+3del mutation implies a deletion that spans 52 base pairs, starting at the position 227094123 and continuing through the position 227094174. Thus, the final 49 base pairs of exon 20 and the first 3 base pairs of intron 20 are removed, and the canonical splice site is disrupted, leading to impaired mRNA splicing. The above schematic representation was made by Achilleas Betsikos and Nikolaos Sabanis through NCBI Genome Data Viewer.

This mutation implies a deletion that spans 52 base pairs, starts at the position 227094123, and continues through the position 227094174 (chr2:227094123-227094174, hg38). More specifically, it removes the final 49 base pairs of exon 20 and the first 3 base pairs of intron 20, disrupting the canonical splice site and therefore impairing normal mRNA splicing (Figure 4).

RNA splicing occurs in two steps: recognition of splice sites, termed canonical, at exon/intron junctions, followed by intron removal and exon ligation. This process is catalyzed by the spliceosome, a complex of five small nuclear ribonucleoproteins and over 300 proteins. Canonical splicing sites begin with the GT base pair in 98.7% of cases, a highly preserved dinucleotide sequence that serves as a marker of proper splicing. Sequences resembling canonical patterns, termed cryptic, may be used when normal splicing is disrupted, as in our case [7].

**Figure 5:**
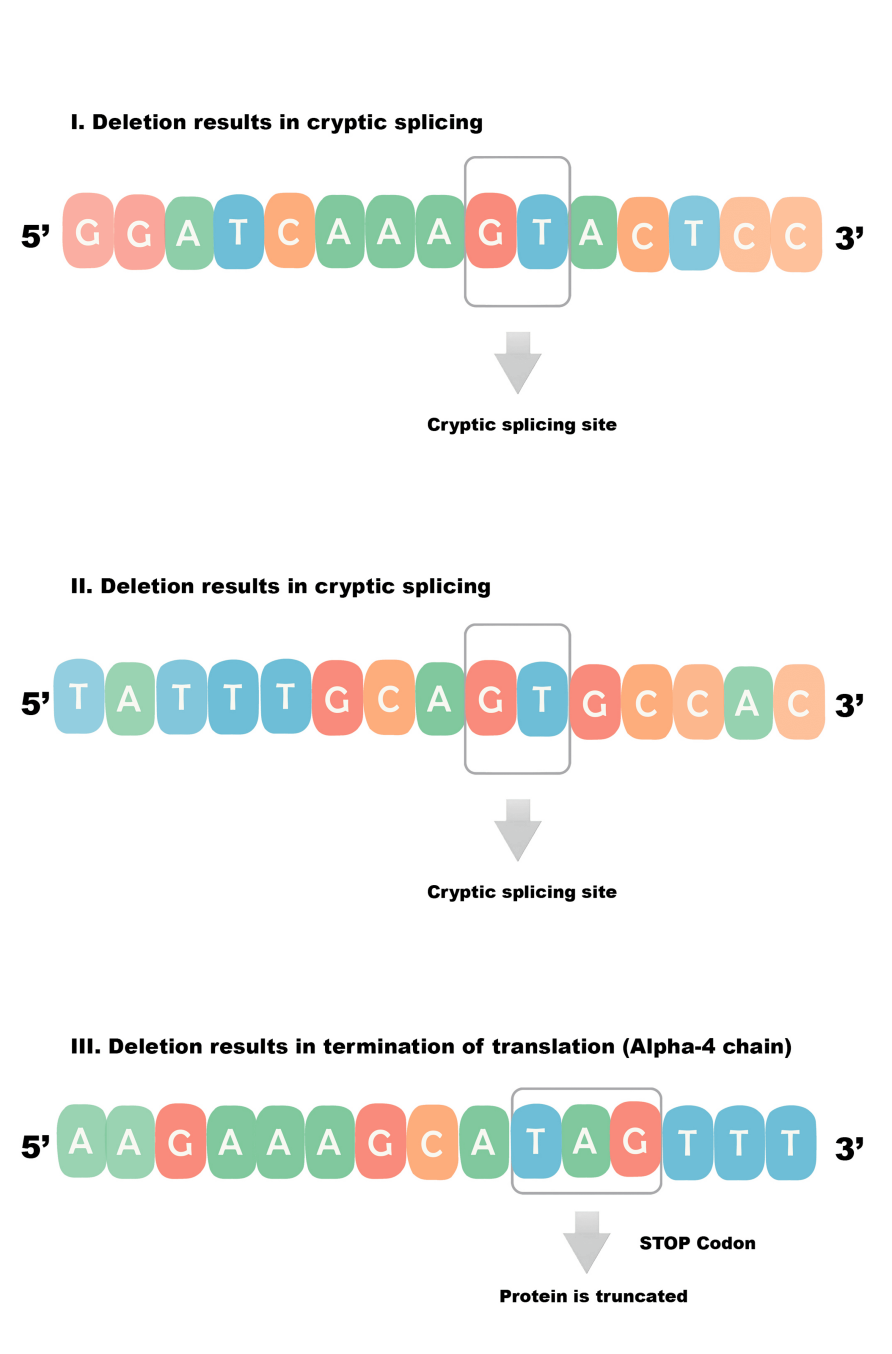
The impact of the c.1321_1369+3del mutation on RNA splicing The impact of the c.1321_1369+3del mutation on RNA splicing revealed a complete loss of the canonical donor site (donor loss score: 1.00, +3 bp) and the emergence of a cryptic donor site nearby. The following scenarios arise: (I) use of a nearby cryptic splice site: the emerging sequence after the deletion leads to the activation of a GT dinucleotide; (II) an alternative cryptic splice site activating a different GT dinucleotide; (III) complete splicing failure, leading to a stop codon and termination of the translation process. The above analysis was made through an in silico study with Splice AI by Achilleas Betsikos and Nikolaos Sabanis.

Using in silico analysis with Splice AI, we explored the impact of the c.1321_1369+3del mutation on RNA splicing. The analysis revealed a complete loss of the canonical donor site (donor loss score: 1.00, +3 bp) and the emergence of a cryptic donor site nearby (donor gain score: 0.81, -2 bp). Four potential scenarios arise: (1) use of a nearby cryptic splice site: the emerging sequence after the deletion leads to the activation of a GT dinucleotide; (2) an alternative cryptic splice site activating a different GT dinucleotide; (3) complete splicing failure, leading to a stop codon and termination of the translation process, thus rendering the final protein truncated; and (4) exon 20 is completely skipped by the spliceosome (Figure 5).

Each scenario points to a significant loss of COL4A4 function, supporting the aforementioned mutation's pathogenic role. Of note, this analysis was performed in silico and therefore carries limitations.

Although previous publications have implicated this particular mutation as a pathogenic variant associated with thin basement membrane disease [8-10], there is no available evidence in the literature linking this mutation to progression to chronic kidney disease (CKD). Herein, we describe a case of an autosomal dominant pattern of inheritance. In line with recent position papers of respected expert panels [2], our finding supports the unified classification of type-IV collagen diseases into a broader spectrum of disease. This case adds another piece to the puzzle in the understanding of the variability of Alport syndrome. To our knowledge, this is the first reported family in which the c.1321_1369+3del mutation is the sole cause of Alport syndrome.

## Conclusions

Alport syndrome is an inherited progressive kidney disease caused by pathogenic variants in genes encoding several members of the collagen type IV protein family. Nowadays, Alport syndrome is considered a primary basement membrane disorder with a broad clinical spectrum associated not only with the pathogenic variant but also the inheritance pattern, which includes, beyond X-linked inheritance, the autosomal dominant and autosomal recessive patterns. In this article, we report a novel pathogenic variant of Alport syndrome following an autosomal dominant pattern of inheritance and highlight the need for accurate genetic diagnosis in cascade screening.
